# Paradoxical upgrading reaction in extra-pulmonary tuberculosis: association with vitamin D therapy

**DOI:** 10.5588/ijtld.16.0927

**Published:** 2017-06

**Authors:** D. A. Barr, A. K. Coussens, S. Irvine, N. D. Ritchie, K. Herbert, B. Choo-Kang, D. Raeside, D. J. Bell, R. A. Seaton

**Affiliations:** * Wellcome Trust Liverpool Glasgow Centre for Global Health Research, University of Liverpool, Liverpool, UK; † Department of Pathology, Faculty of Health Sciences, Institute of Infectious Diseases and Molecular Medicine, University of Cape Town, South Africa; ‡ Institute of Infection, Immunity and Inflammation, College of Medical, Veterinary and Life Sciences, University of Glasgow, Glasgow, Scotland, UK; § Hairmyres Hospital, East Kilbride, Scotland, UK; ¶ Department of Respiratory Medicine, Glasgow Royal Infirmary, Glasgow, Scotland, UK; # Departments of Respiratory Medicine, Scotland, UK; ** Infectious Diseases, The Queen Elizabeth University Hospital, Glasgow, Scotland, UK

**Keywords:** host-directed therapy, innate immunity, host–pathogen interaction, inflammation

## Abstract

**SETTING:**

Glasgow, Scotland, UK.

**BACKGROUND:**

Paradoxical reactions in tuberculosis (TB) are a notable example of our incomplete understanding of host-pathogen interactions during anti-tuberculosis treatment.

**OBJECTIVES:**

To determine risk factors for a TB paradoxical reaction, and specifically to assess for an independent association with vitamin D use.

**DESIGN:**

Consecutive human immunodeficiency virus (HIV) negative adult patients treated for extra-pulmonary TB were identified from an Extended Surveillance of Mycobacterial Infections database. In our setting, vitamin D was variably prescribed for newly diagnosed TB patients. A previously published definition of paradoxical TB reaction was retrospectively applied to, and data on all previously described risk factors were extracted from, centralised electronic patient records. The association with vitamin D use was assessed using multivariate logistic regression.

**RESULTS:**

Of the 249 patients included, most had TB adenopathy; 222/249 had microbiologically and/or histologically confirmed TB. Vitamin D was prescribed for 57/249 (23%) patients; 37/249 (15%) were classified as having paradoxical reactions. Younger age, acid-fast bacilli-positive invasive samples, multiple disease sites, lower lymphocyte count and vitamin D use were found to be independent risk factors.

**CONCLUSION:**

We speculate that vitamin D-mediated signalling of pro-inflammatory innate immune cells, along with high antigenic load, may mediate paradoxical reactions in anti-tuberculosis treatment.

## RESUME

**OBJECTIFS:**

Les réactions paradoxales à la tuberculose (TB) sont un exemple notable de notre compréhension incomplète de l'interaction hôte pathogène pendant le traitement de la TB. Nous avons voulu déterminer les facteurs de risque de réaction paradoxale à la TB et spécifiquement évaluer une association indépendante avec la prescription de vitamine D.

**SCHÉMA:**

Des patients adultes consécutifs négatifs à l'infection pour le virus de l'immunodéficience humaine (VIH) traités pour une TB extrapulmonaire ont été identifiés à partir d'une base de données de Surveillance Etendue des Infections à Mycobactéries. Dans notre contexte, la vitamine D est prescrite de manière variable aux patients TB nouvellement diagnostiqués. Une définition précédemment publiée des réactions paradoxales à la TB a été rétrospectivement appliquée aux dossiers électroniques centralisés des patients et les données de tous les facteurs de risque précédemment décrits ont été extraites de ces dossiers. L'association de la prescription de vitamine D a été évaluée par régression logistique multivariable.

**RÉSULTATS:**

La majorité des 249 patients inclus avait des adénopathies TB ; 222/249 avaient une TB confirmée par microbiologie et/ou par histologie. La vitamine D a été prescrite à 57/249 (23%) patients ; 37/249 (15%) ont été classés comme ayant des réactions paradoxales. Les facteurs de risque indépendants trouvés ont été le jeune âge, un échantillon positif aux bacilles acido-alcoolo-résistants, de multiples sites de maladie, une numération de lymphocytes plus faible et la prescription de vitamine D.

**CONCLUSION:**

Nous spéculons que les cellules immunitaires pro-inflammatoires innées médiées par la vitamine D, avec une charge antigénique élevée, pourraient être à l'origine des réactions paradoxales pendant le traitement de la TB.

## RESUMEN

**MARCO DE REFERENCIA:**

La ciudad de Glasgow en Escocia, en el Reino Unido.

**OBJETIVOS:**

La reacción paradójica al tratamiento antituberculoso representa un claro ejemplo de la deficiencia de los conocimientos sobre las interacciones entre el agente patógeno y el hospedero durante el tratamiento de la tuberculosis (TB). El estudio buscó determinar los factores de riesgo de aparición de la reacción paradójica y examinar de manera específica una asociación con la administración de vitamina D.

**MÉTODO:**

Se escogieron de la base de datos de la vigilancia ampliada de las infecciones por micobacterias los pacientes adultos consecutivos, negativos frente al virus de la inmunodeficiencia humana (VIH), tratados por TB extrapulmonar. En este entorno se receta de manera variable la vitamina D a los pacientes con diagnóstico reciente de TB. Se utilizó la definición de reacción paradójica al tratamiento antituberculoso publicada en un artículo anterior y se extrajeron de las historias clínicas informáticas centralizadas los datos relacionados con todos los factores de riesgo conocidos. Mediante un análisis de regresión logística se evaluó el efecto de la administración de vitamina D.

**RESULTADOS:**

Se incluyeron en el estudio 249 pacientes; la mayoría presentó adenopatía tuberculosa; en 222/249 casos hubo confirmación microbiológica o patológica del diagnóstico de TB. Se recetó la vitamina D a 57/249 pacientes (23%) y se diagnosticó la reacción paradójica en 37 casos (15%). Los factores de riesgo independientes revelados por el estudio fueron una edad más temprana, la presencia de bacilos acidorresistentes en una muestra obtenida mediante una técnica cruenta, la enfermedad con localizaciones múltiples, un recuento más bajo de linfocitos y la administración de vitamina D.

**CONCLUSIÓN:**

Se formula la hipótesis de que la vitamina D actúa como mediador de la señalización celular de la inmunidad innata proinflamatoria y que, asociada con una gran carga antigénica, puede participar en las reacciones paradójicas durante el tratamiento antituberculoso.

WORSENING OF TUBERCULOSIS (TB) disease despite receipt of effective anti-tuberculosis treatment is referred to as a ‘paradoxical upgrading reaction’ (PUR). A PUR is a clinical diagnosis based on worsening of an existing TB lesion or development of newly apparent TB lesions, which are typically culture-negative and not associated with treatment failure.^1^–^7^ PURs are most frequently diagnosed at extra-pulmonary sites, where they can cause significant morbidity. In addition, imaging modalities such as positron emission tomography-computed tomography reveal that most pulmonary TB (PTB) patients have new lesions or lesions with increased metabolic activity after 6 months of anti-tuberculosis treatment despite sputum culture conversion.^8^ Similar rates of new, subclinical lesions are seen on serial magnetic resonance imaging of the brains of patients after receipt of treatment for central nervous system TB.^9^ Rather than being an unusual event, PURs may be an underappreciated central feature of the interaction between Mycobacterium tuberculosis, host immunity and antimicrobial treatment.

Historically, PURs have been thought to be analogous to ‘upgrading reactions’ in leprosy.^10^ More recently, TB PUR in the context of human immunodeficiency virus (HIV) associated immune reconstitution inflammatory syndrome (TB-IRIS) has been described. Pathogenesis of antiretroviral treatment-associated IRIS is increasingly understood to involve innate immune mediators, including Toll-like receptor (TLR) signalling.^11^

How a PUR might develop in the absence of overt reversal of immune suppression as observed in HIV-associated IRIS is not clear. A small number of studies have indicated extra-pulmonary TB (EPTB), baseline lymphopaenia and increased peripheral lymphocyte reconstitution to be risk factors in non-HIV-infected TB patients.^12^,^13^ Vitamin D is a known immune modulator in TB infection,^14^ and vitamin D deficiency has been associated with PURs in some case reports.^13^ No studies have assessed vitamin D supplementation as a modulator of PUR risk. A randomised control trial of high-dose vitamin D supplementation during the intensive phase of PTB treatment reported a PUR in 2/71 in the intervention group and 0/70 in the placebo group—a non-significant difference.^15^ However, that study had 56 days of follow-up (less than the median time to a PUR in most studies) and was not powered to detect PUR outcomes. A potential association between use of vitamin D and PURs is therefore a pressing but open question.^16^

Prescription of vitamin D is increasingly common practice in TB clinics in our setting. This gave us an opportunity to carry out a retrospective cohort study to examine the effect of vitamin D use on the risk of symptomatic PURs in patients treated for EPTB.

## METHODS

Patients treated for EPTB at four hospitals collectively responsible for >95% of TB management in the Greater Glasgow and Clyde area of Scotland were included in a retrospective cohort. In this setting, vitamin D was prescribed ad hoc such that patients received vitamin D at the discretion of individual treating clinicians. Included were all consecutive patients who: 1) were aged ⩾18 years; 2) had standard therapy for confirmed EPTB (culture-positive or positive acid-fast bacilli [AFB] histology or smear) or probable EPTB (clinical, histological or radiological evidence of TB with response to anti-tuberculosis treatment); and 3) were HIV-negative. Patients already prescribed vitamin D before the diagnosis of TB, and those with <3 months of recorded follow-up, were excluded.

Patients were identified from an Extended Surveillance of Mycobacterial Infections database to which all TB cases in Glasgow are notified. Demographic and clinical data were obtained from centralised electronic patient records and patient folders as necessary. Independent variables collected included all previously published risk factors for a PUR, and any prescription of a vitamin D supplement during TB treatment as a binary variable (for variable definitions and prior literature review, see the Appendix).^*^ This retrospective review of routinely collected data was exempted from formal ethics review.

A published definition of a PUR^1^ (‘worsening of pre-existing tuberculous lesions on the basis of clinical or radiological findings or development of new TB lesions in patients who had received anti-tuberculosis treatment for at least 10 days and whose conditions were reported to be improving’) was retrospectively applied to all cases by two consultants in infectious diseases (RAS and DJB) blinded to each other's assessment and the status of vitamin D prescription. In cases of disagreement, a third independent application of the case definition (DAB) was used to break ties.

Assuming vitamin D was prescribed in 50% of EPTB patients with an overall PUR prevalence of 18%, a sample size of 250 patients was calculated to give 0.80 power at α = 0.05 to detect a 12% absolute increase in PURs associated with vitamin D use. Pairwise comparison of variables was by Fisher's exact test for categorical variables, and Wilcoxon's rank-sum test for non-normal numerical variables. The association of vitamin D use with a PUR after adjustment for variables thought to be potential confounders was assessed by logistic regression. All statistical analyses were carried out in R Studio v0.99.902 (R Computing, Vienna, Austria); R code to reproduce this analysis is available at https://github.com/davidadambarr/PUR.EPTB.VitD.

## RESULTS

### Description of cohort

Of 260 patients included, 249 were assessed; 11 were excluded due to evidence of vitamin D prescription before a TB diagnosis (*n* = 5) or due to <3 months of recorded follow-up (*n* = 6). Basic demographic and clinical descriptors are shown in Table 1. Most patients were of South Asian ethnicity; lymph nodes were the most common site of disease. Invasive diagnostic sampling (e.g., biopsy or aspiration) was attempted in 230 patients (92%): 153/230 (67%) were M. tuberculosis culture-positive; 69/230 (30%) were culture-negative but AFB-positive or had histological features in keeping with TB disease.

**Table 1:**
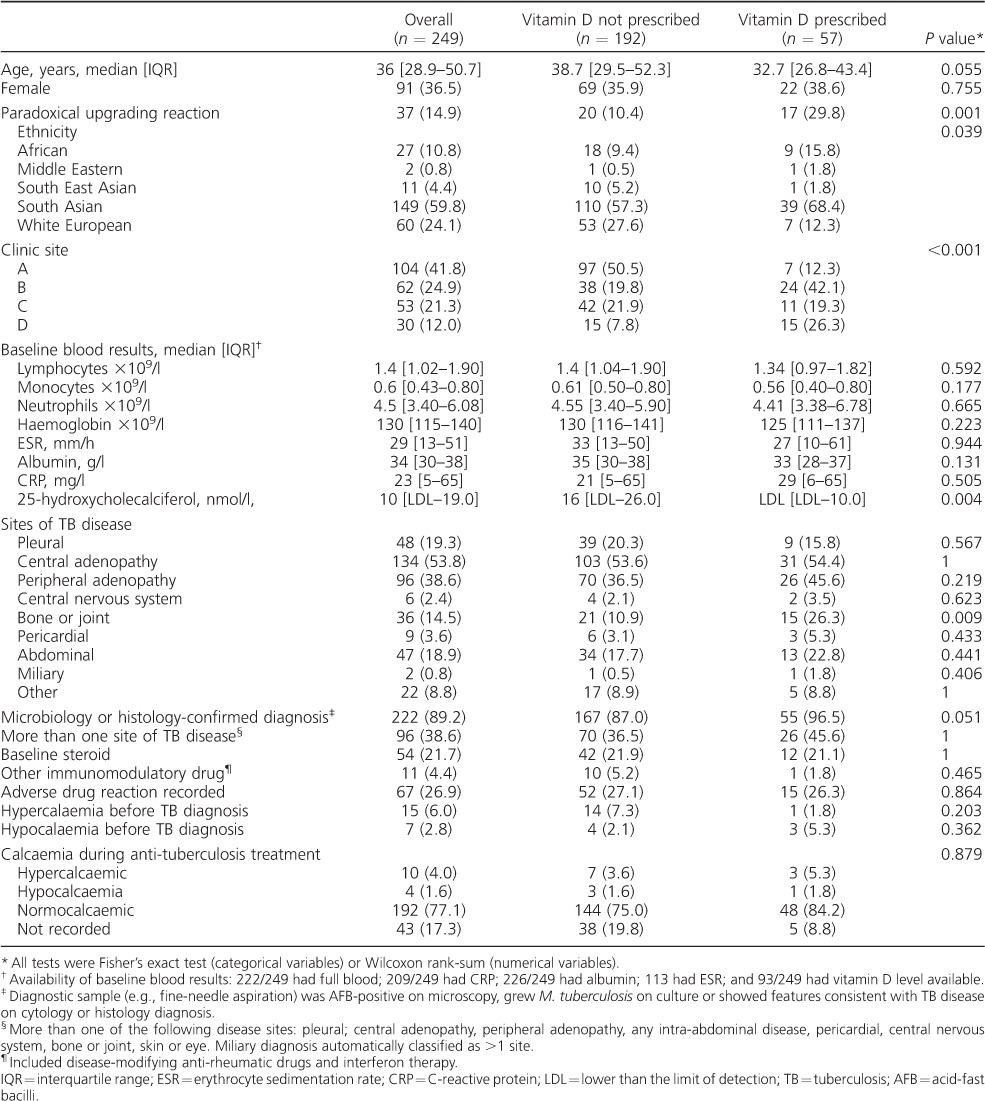
Description of cohort

Of the 93 patients (37%) who had a baseline serum level of 25-hydroxycholecalciferol checked, 45/93 (48%) had levels below the limit of detection for the assay (<7 nmol/l, range <7 to 114 nmol/l). Vitamin D was prescribed for 57 patients (23%), not necessarily according to baseline status, because not all patients prescribed vitamin D had a baseline level checked. For 52 (91%) of these patients, a dose equivalent to ⩽800 international units (IU) of colecalciferol per day was used, and 5/57 (9%) received a one-off dose of 300 000 IU, followed by 20 000 IU monthly. Prescription of vitamin D differed according to patient ethnicity, the clinic at which TB was being treated, and by the baseline level of vitamin D, although vitamin D deficiency was also prevalent among patients not prescribed vitamin D (Table 1).

Thirty-seven patients (15%) were classified as having a PUR. Inter-rater agreement was high for the two primary assessors (Cohen's κ 0.84; 95% confidence interval [CI] 0.74–0.94). The time of the first PUR after starting anti-tuberculosis treatment had a positively skewed distribution (median 52 [range 10–500] days). Corticosteroid treatment was prescribed for 14/37 (38%) patients after a PUR; 5/37 (14%) had percutaneous drainage; 12/37 required no specific treatment; and one patient had surgical intervention for constrictive pericarditis associated with a PUR.

Of 249 patients, 241 (97%) had a recorded outcome available at the end of the treatment: 239/241 (99%) were recorded as ‘clinically cured’, the remaining two died on treatment (neither thought to be related to a PUR). A median post-treatment follow-up of 12 months was recorded; 3/239 (1%) patients had recorded recurrent/relapsed TB at respectively 2, 4 and 24 months after the end of treatment.

### Univariate associations with a paradoxical upgrading reaction

Variables associated with a PUR on univariate testing were lower age, the clinic site where treatment was given, lower lymphocyte count, having an AFB-positive diagnostic sample at baseline, having more than one site of TB disease at baseline and being prescribed vitamin D during anti-tuberculosis treatment (Table 2).

**Table 2:**
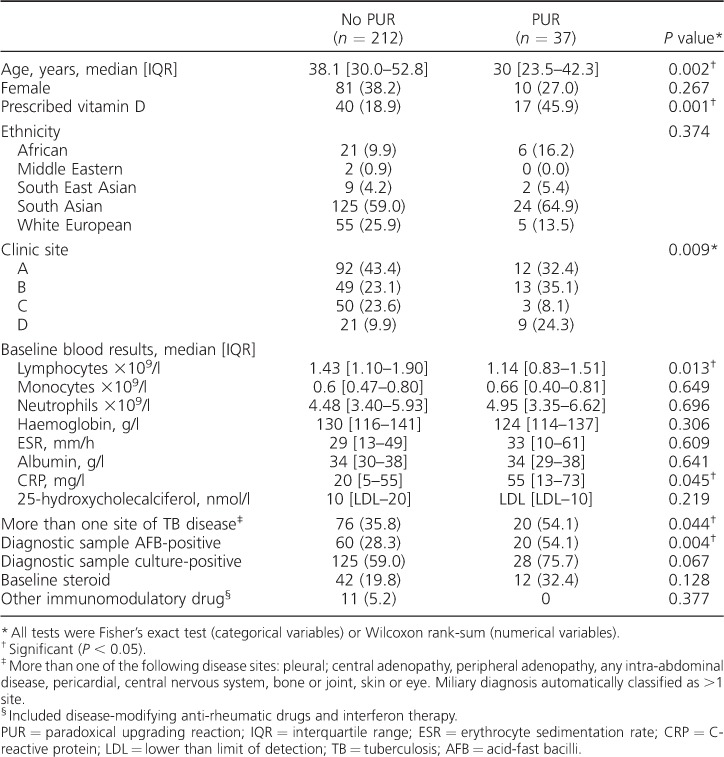
Univariate associations with a PUR

### Multivariate associations with a paradoxical upgrading reaction

Although ethnicity was not significantly associated with a PUR (*P* = 0.374), it was considered to be a potential confounder because prescribing of vitamin D was influenced by patient ethnicity (Table 1, *P* = 0.039). The association between prescription of vitamin D and a PUR was adjusted for ethnicity in a logistic regression model, and remained significant, with an odds ratio (OR) of 3.35 (95%CI 1.59–7.06, *P* = 0.001; see Appendix http://rpubs.com/davidadambarr/EPTB-PUR-VitD). When the ethnicity variable was collapsed into three categories—white European, African, Asian (including Middle Eastern, South East Asian and South Asian) due to low frequencies in some of the pre-specified ethnic categories—prescription of vitamin D was associated with a higher rate of PUR in each grouping (Figure A).

**Figure:**
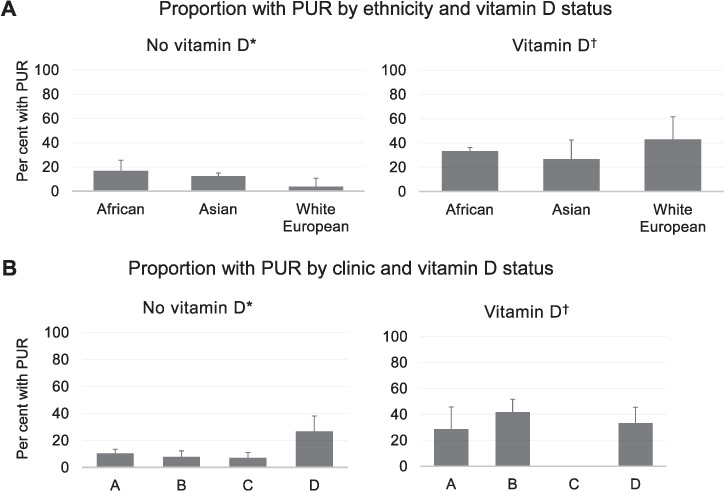
**A)** Patient ethnicity and **B)** clinic site as possible confounders of association between vitamin D and a paradoxical upgrading reaction. Error bars are standard errors for the proportion based on binomial probability distribution. *No prescription of vitamin D during anti-tuberculosis treatment. ^†^Vitamin D supplement prescribed during anti-tuberculosis treatment.

The clinic site was also considered an important potential confounder because clinics had different rates of vitamin D prescribing and different rates of observed PURs. Clinic C was found to be an outlier with much lower rates of vitamin D prescribing and PUR than the other sites. Only 11/53 (21%) of patients at clinic C were prescribed vitamin D, and none had an observed PUR (Figure B). This ‘zero frequency cell’ in a contingency table of a PUR by clinic site and vitamin D prescription meant that clinic site could not be included in a full multivariate model. Instead, an exact logistic regression model^17^ was performed to adjust vitamin D prescription for clinic site. In this model, vitamin D prescription remained significant, with an OR of 3.70 (95%CI 1.54–13.23, *P* < 0.001; see Appendix http://rpubs.com/davidadambarr/EPTB-PUR-VitD). In addition, each patient prescribed vitamin D was matched with a control using a propensity score for vitamin D prescription based on all the variables associated with vitamin D prescription and a PUR (clinic site, age and ethnicity). In this analysis, patients prescribed vitamin D remained at greater risk for a PUR than propensity score-matched controls (OR 2.60, 95%CI 1.04–6.96, *P* = 0.046; see Appendix http://rpubs.com/davidadambarr/EPTB-PUR-VitD).

Finally, to create a multivariate model, all variables found to be significant on univariate testing except the clinic site were used. Ethnicity was also included as a potential confounder. In this ‘full’ model, younger age, an AFB-positive diagnostic sample, lymphocyte count and vitamin D prescription had largely unchanged OR estimates (Table 3). A more parsimonious model with variables deselected stepwise based on the Akaike Information Criterion was found to have equivalent fit and predictive performance. This model retained age, AFB status of the diagnostic sample, lymphocyte count, multiple sites of TB disease at baseline and vitamin D prescription as important independent variables (Table 3).

**Table 3:**
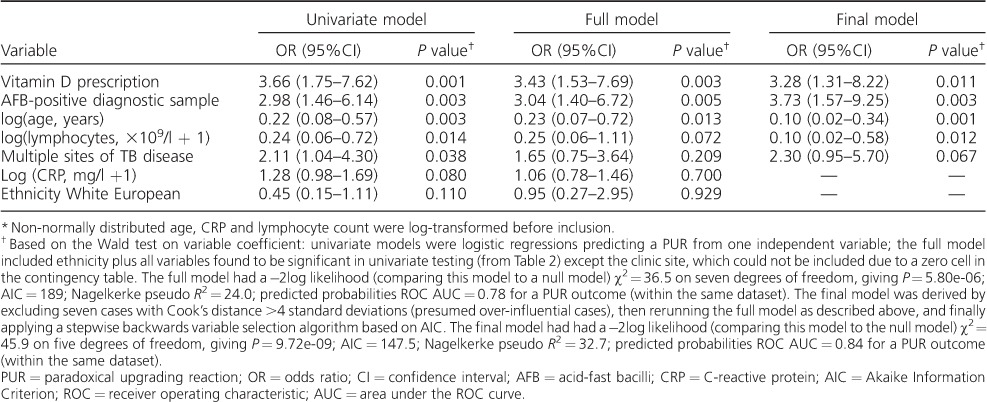
Logistic regression results for associations with a PUR ^*^

## DISCUSSION

This is the first cohort study to examine the relationship between use of vitamin D and the risk of a PUR. We found a significant independent increased risk of a PUR to be associated with younger age, AFB positivity of the diagnostic sample, lymphopaenia, multiple sites of TB disease and receipt of vitamin D supplementation at baseline.

Younger age^2^,^4^,^18^–^20^ and lower lymphocyte count^5^,^19^,^21^,^22^ at the time of the TB diagnosis have been identified as risk factors for a PUR in previous cohort studies, and are mechanistically plausible mediators of the host immune response in a PUR. We also found having an AFB-positive diagnostic sample and multiple sites of TB disease at diagnosis to be risk factors for a PUR. Patients in this cohort were extensively investigated—92% underwent invasive sampling to attempt a microbiological or histological diagnosis before treatment—thereby reducing the risk of bias in these estimates. Several studies have found more extensive disease at baseline to be a risk factor,^3^,^18^,^22^ and a trend towards higher PUR in AFB-positive cases has also been described.^1^ More extensive disease and AFB-positive diagnostic sample variables suggest that a higher baseline antigen load is related to PUR development.

The active metabolite, 1α,25-dihydoxy vitamin D (1α,25(OH)2D3), supports an innate pro-inflammatory TLR-associated macrophage response in vitro, a response necessary for effective intracellular mycobacterial killing.^23^ Pre-treatment of monocytes with 1α,25(OH)2D3 induces cellular maturation and increased production of the innate cytokine tumour necrosis factor following lipopolysaccharide stimulation via TLR4 signalling.^24^ Mycobacterial stimulation of TLR1/2 on monocytes also leads to enhanced expression of the vitamin D receptor and 1α-hydroxylase CYP27B1 and, in the presence of sufficient vitamin D, leads the antimicrobial activity via cathelicidin production.^23^ Enhancement of TLR signalling by supplementation with vitamin D in a patient with deficiency of vitamin D could therefore plausibly cause an upgraded innate immune response analogous to that seen in TB-IRIS.

Conversely, the effects of vitamin D on the adaptive immune system are thought to be anti-inflammatory, driving FoxP3 and CTLA4 expression, markers of regulatory T (Treg) cells and promoting type 2 T helper (Th2) cells, and blocking production of pro-inflammatory cytokines interleukin (IL) 2, IL-17 IL-21 and interferon-gamma.^25^,^26^ However, these observations vary according to the timing of treatment, the differentiation status of the vitamin D-treated cells and the presence of microbial products during treatment. Naïve CD4^+^ T-cells treated with active vitamin D suppress IL-4 production (the hallmark of Th2 cells), whereas co-treatment of CD4^+^ and CD8^+^ T cells with 1α,25(OH)2D3 and IL-4 induces IL-6 production.^26^,^27^

Thus, vitamin D supplementation, depending on immune status and degree of antigen load,^28^ might just as much uncover or exacerbate pathological immune imbalances specific to some TB-susceptible hosts as it may prevent or resolve them. We speculate that supplementation of at-risk patients with high antigen load may lead to exacerbation of the innate TLR-mediated response, leading to exacerbated innate cytokine signalling similar to that observed in TB-IRIS. This response is also analogous to a reversal reaction in leprosy (progression from lepromatous to tuberculoid leprosy), and is associated with a switch in the inflammatory balance from phagocytic to vitamin D-mediated antimicrobial macrophage function and clearance of mycobacteria.^29^ A reversal reaction is also associated with increased FoxP3 staining in lesions,^30^ consistent with the role of vitamin D in Treg differentiation, as well as influx of Th1 cells.^31^

The activation of the innate response by vitamin D primarily induces antimicrobial responses; a PUR could be associated with improved clearance of bacteria, but at the cost of increased inflammatory disease. This hypothesis suggests that the role of host-directed therapies (HDTs) such as vitamin D should be defined according to the specific clinical problem they are intended to solve, and that their effects may differ across the spectrum of TB disease and host response. Reassuringly, and as shown previously, a PUR was not associated with a high risk of serious adverse outcomes in our cohort, but did cause significant morbidity for some patients. Microbiological failure rates in EPTB cannot be routinely determined, but failures in clinical treatment or relapses in our low HIV, low multidrug-resistant TB setting were <2%. To gain maximum utility from future HDTs, knowing which patients can benefit from enhanced bactericidal activity and which could benefit from anti-inflammatory therapy is necessary.

Weaknesses of this study stem from its retrospective design. The definition of a PUR had to be applied retrospectively due to differences between treating clinicians in how formally these cases were diagnosed. Inter-rater agreement was, however, strong despite this limitation. The four clinic sites used in this study had markedly different patient characteristics. However, as the clinic site could not be included in the final multivariate modelling due to the low frequency of events and vitamin D prescription at one clinic, this potential confounding could not be addressed fully. Finally, too few patients had serial serum levels of vitamin D checked to allow direct analyses of dose–concentration and PUR-response relationships, which would have provided a more robust test of a causal relationship between vitamin D and PURs, as would the measurement of vitamin D-associated inflammatory markers.

## CONCLUSIONS

The PUR phenomenon is further evidence that, despite our current standardised approach to treatment, TB disease exists in a spectrum of host–pathogen interactions. We should anticipate that the effects of future HDTs may be stratified by patient variables such as age, vitamin D status, lymphocyte count, antigenic load and inflammatory status. Most importantly, our results highlight that future trials of HDTs should consider adequate powering to detect PUR outcomes and prospectively define patient subgroups who may respond differently to these novel therapies.

## Figures and Tables

**Table A.1:**
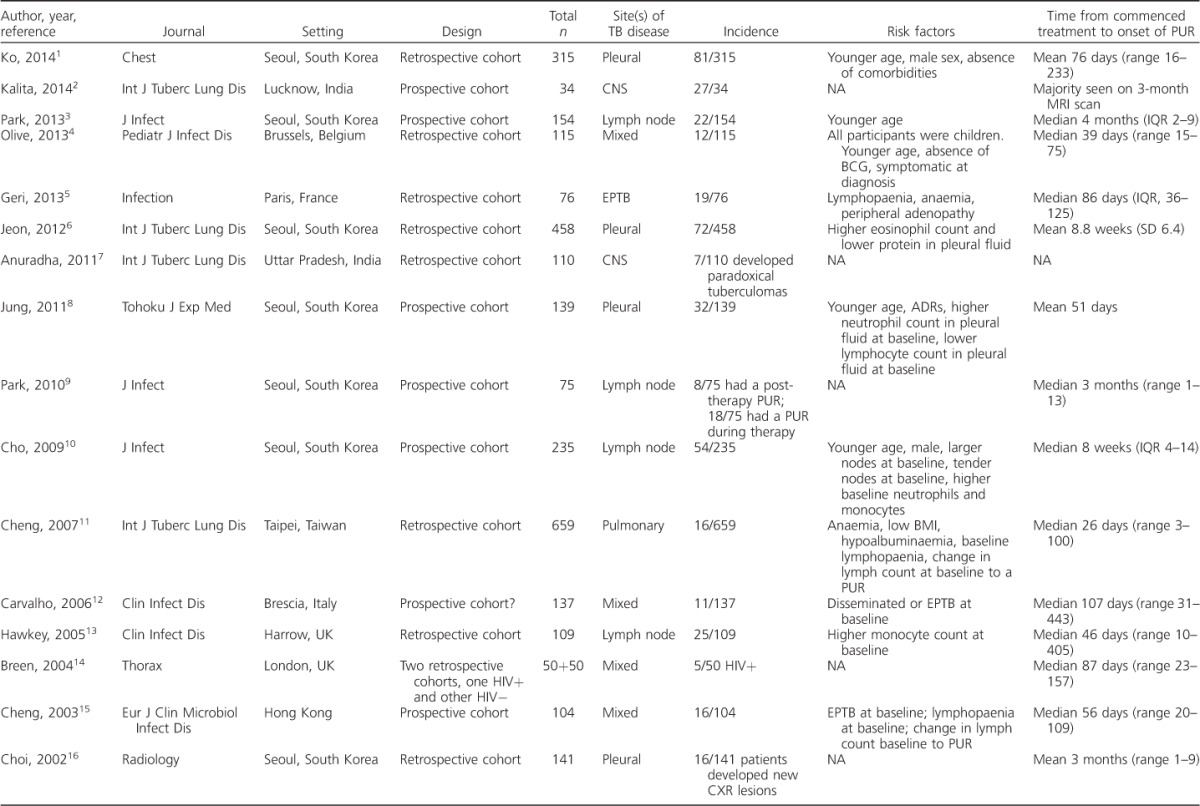
Identified through PubMed search (Tuberculosis[MeSH Terms]) AND (upgrading reaction[Title/Abstract] OR paradoxical[Title/Abstract])

**Table A.1:**
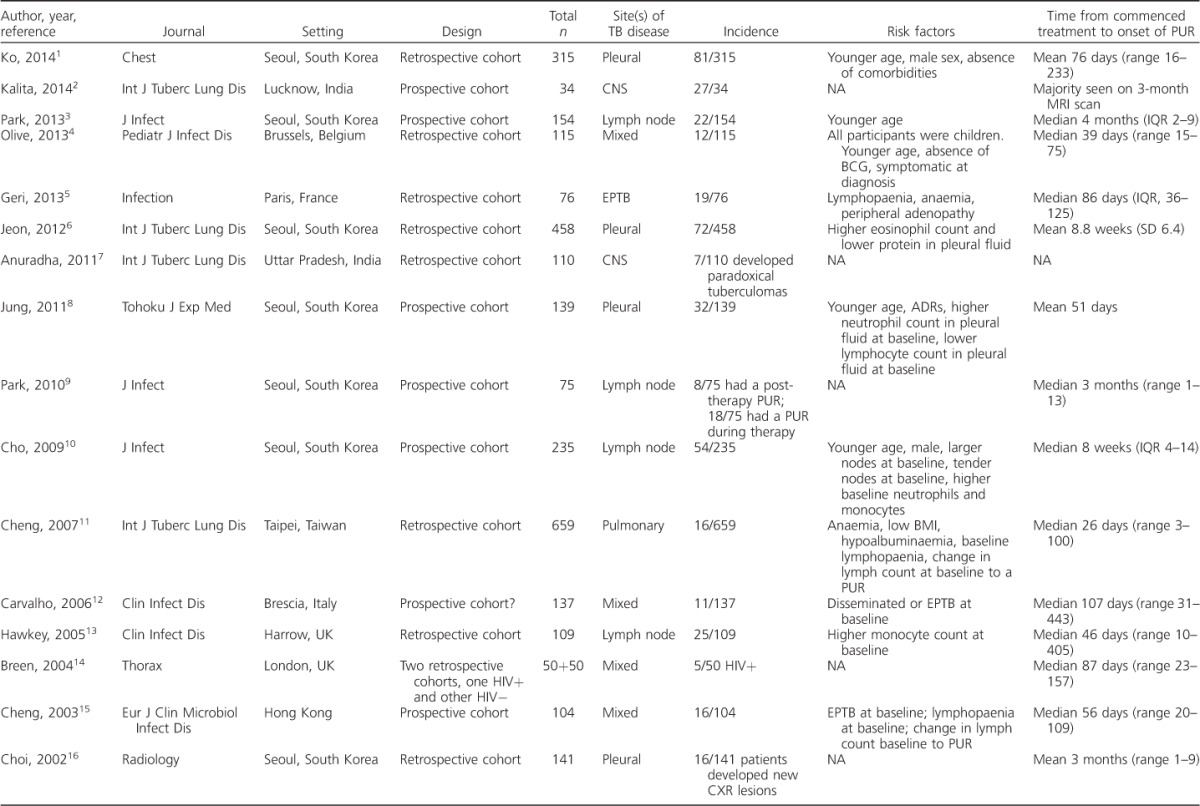
(Continued)

**Table A.2:**
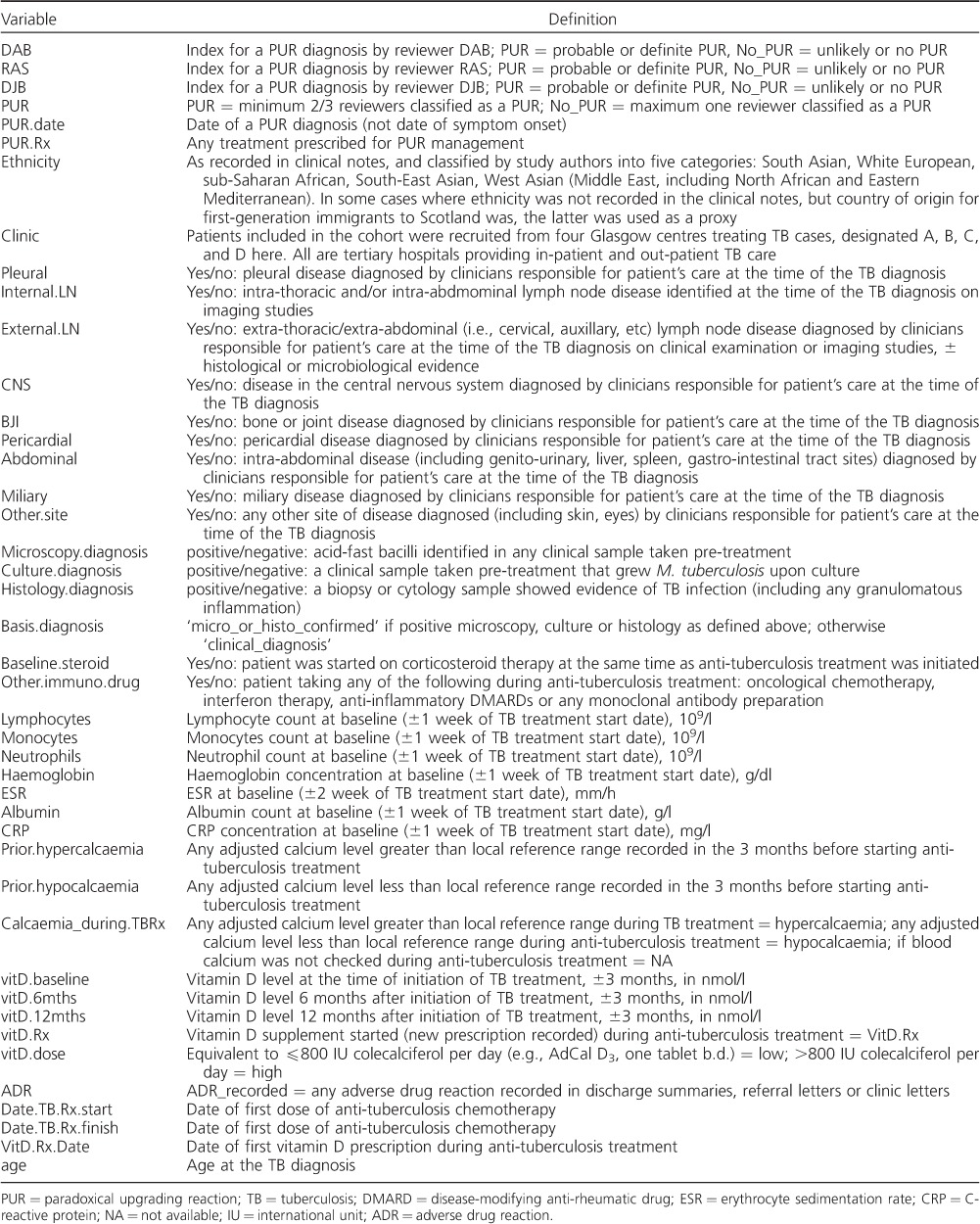
Definitions of variables in raw data

## Footnotes

^*^ The appendix is available in the online version of this article, at http://www.ingentaconnect.com/content/iuatld/ijtld/2017/00000021/00000006/art00013
